# The Hospital World

**Published:** 1911-01-14

**Authors:** 


### THE HOSPITAL WORLD.

### EDINBURGH ROYAL INFIRMARY.

We take the following from the Annual Report of the Royal Infirmary, Edinburgh, which has just been issued for 1910: "The managers deplore the death of two former members of the board, to whom the Royal Infirmary owes much—Lord Pearson, a member from 1900 to 1904, and Mr. Patrick Blair, W.S., a member from 1894 to 1908. It was owing to the initiative of Lord Pearson that the out-patients' departments were reorganised, while to Mr. Blair the Royal Infirmary is indebted, not only for long and arduous service as chairman of the House and Finance Committees, but more especially for the establishment of the Nurses' Home of Rest at Colinton, which as 'Blair House' perpetuates his name."

The report goes on to praise the work of two members of the board, who, having completed their five years' tenure of office, retired at the end of December: "One is Lord Salvesen, who has represented the Senators of the College of Justice, and has taken a large share in the work of the board, acting as convener of a number of special committees with much acceptance to his fellow-managers. The other is Mrs. Kerr, one of the representatives of the Court of Contributors. Mrs. Kerr has rendered invaluable service to the institution, more especially during the time when she has been convener of the Nursing Committee. She has spared herself neither time nor trouble in becoming acquainted with the requirements of the Infirmary; and her capable and sympathetic work in connection with the nursing and serving departments has been peculiarly appreciated. The managers, in no ordinary degree, will miss her presence at their meetings."

At the recent annual meeting of the Edinburgh Royal Infirmary, Lord Salvesen, who presided, took the opportunity of paying to two of the officials a high tribute, which we note: "Our treasurer and clerk," said his lordship "has just completed thirty years' service under the Infirmary Board. (Applause.) I am glad to say that Mr. Caw is with us still, and hope he will be for many years to come. He is in the full strength of his activities, and the experience he has derived from thirty years' service is an invaluable asset to the infirmary. It is interesting to see what has been the progress of the institution during these thirty years. In 1881 the number of indoor patients who were treated in the infirmary was 5,746. In 1910 the number had increased to 12,342; and since 1881 the outdoor patients have increased from 15,000 to 38,960.

"There is another official connected with the infirmary who, I am sorry to say, has thought it necessary, from failing health and advancing years, to tender his resignation. I refer to Colonel Warburton, who for over ten years has now been superintendent of the infirmary. Colonel Warburton entered upon his duties in the autumn of the year 1899, and came with a high reputation from India, where he had served for many years in many important positions. During the last four years he had held the office of Inspector-General of Civil Hospitals of the United Provinces of Agra and Oude, and during his term of office there the Provinces suffered from a widespread famine and were threatened with a serious attack of plague. It was due to Colonel Warburton's systematic measures and complete organisation that the plague, which had already broken out in a corner of the North-West Provinces, was effectualy prevented from spreading any further, and he had the highest testimonials from the governors of these provinces and other leading officials in India when he came to Edinburgh. Colonel Warburton has more than justified the very high expectations formed of him when he entered the office. I will offer only one example to give a notion of how much may be done by a zealous and efficient official occupying a position of importance such as Colonel Warburton has done. In the first year of his service the expenditure per bed was £68 3s. 8d. In the year now under review the expenditure had been reduced to £64 17s. 10d. That was in itself a considerable saving, when we reflect on what had been the general trend of prices during the past thirty years. But it would not be fair to Colonel Warburton to represent that as all, because during the past thirty years there has been thrown upon the infirmary a very large annual expenditure for the purpose of improving not merely the position of the nurses but the position of the patients. For instance, as regarded nurses. During his term of office the unpaid probationers were replaced by paid probationers, and the nurses had been secured in pension rights which they did not enjoy ten years ago." (Applause.)

